# The efficacy of oral azithromycin in clearing ocular chlamydia: Mathematical modeling from a community-randomized trachoma trial

**DOI:** 10.1016/j.epidem.2013.12.001

**Published:** 2014-01-08

**Authors:** Fengchen Liu, Travis C. Porco, Harran A. Mkocha, Beatriz Muñoz, Kathryn J. Ray, Robin L Bailey, Thomas M. Lietman, Sheila K. West

**Affiliations:** a F.I. Proctor Foundation, University of California, San Francisco, CA, USA; b Department of Ophthalmology, University of California, San Francisco, CA, USA; c Department of Epidemiology & Biostatistics, University of California, San Francisco, CA, USA; d Kongwa Trachoma Project, Kongwa, Tanzania; e Faculty of Infectious and Tropical Diseases, Clinical Research Department, London School of Hygiene & Tropical and Medicine, London, UK; f Institute for Global Health, University of California, San Francisco, CA, USA; g Wilmer Eye Institute, Johns Hopkins Hospital, Baltimore, MD, USA

**Keywords:** Mathematical model, Elimination, Azithromycin, SAFE strategy, Trachoma

## Abstract

Mass oral azithromycin distributions have dramatically reduced the prevalence of the ocular strains of chlamydia that cause trachoma. Assessing efficacy of the antibiotic in an individual is important in planning trachoma elimination. However, the efficacy is difficult to estimate, because post-treatment laboratory testing may be complicated by nonviable organisms or reinfection. Here, we monitored ocular chlamydial infection twice a year in pre-school children in 32 communities as part of a cluster-randomized clinical trial in Tanzania (prevalence in children was lowered from 22.0% to 4.7% after 3-year of annual treatment). We used a mathematical transmission model to estimate the prevalence of infection immediately after treatment, and found the effective field efficacy of antibiotic in an individual to be 67.6% (95% CI: 56.5–75.1%) in this setting. Sensitivity analyses suggested that these results were not dependent on specific assumptions about the duration of infection. We found no evidence of decreased efficacy during the course of the trial. We estimated an 89% chance of elimination after 10 years of annual treatment with 95% coverage.

## Introduction

The World Health Organization (WHO) has targeted trachoma for elimination by the year 2020 (Mariotti, 2004). Repeated mass oral azithromycin distribution is a central component of the SAFE (Surgery of trichiasis, Antibiotics, Facial cleanliness and Environmental improvement) strategy endorsed by the WHO. Theoretically, repeated treatments may eventually eliminate infection from even the most severely affected areas (Lietman et al., 1999; Melese et al., 2004), and mass antibiotic distributions have, in fact, dramatically reduced the prevalence of infection in a number of locations (Burton et al., 2010; Chidambaram et al., 2006; Gaynor et al., 2003; House et al., 2009; Melese et al., 2004; Schachter et al., 1999; Solomon et al., 2004; West et al., 2005). However, concern remains that chlamydia may develop resistance to the azalides and macrolides, and that azithromycin may lose efficacy over time. *In vitro* resistance has not been observed, although it is difficult to assess and rarely tested. Small surveys after one and after four mass azithromycin distributions have failed to find drug resistance (Hong et al., 2009; Solomon et al., 2005).

The efficacy of repeated oral azithromycin distributions has been reported at the community level (Gaynor et al., 2003; Gebre et al., 2012; Melese et al., 2008; Schachter et al., 1999). However, the efficacy in an individual (probability of clearance following treatment) has been difficult to assess; treated individuals may become infected between pre-treatment and post-treatment examinations (which may be as much as 6 months) even in carefully monitored communities. Although the true probability of clearance following treatment cannot fully be assessed under field conditions because of reinfection and false positivity due to dead organisms immediately after treatment, analysis of longitudinal prevalence during trachoma elimination programs nevertheless reveals profound reductions in prevalence during treatment, as described elsewhere (Chidambaram et al., 2006; Melese et al., 2004; Solomon et al., 2004). Since these reductions occur because of the efficacy of the antibiotic in eliminating infection from individuals, analysis of such longitudinal prevalence curves reveals information about the efficacy. Lowered values for the individual efficacy correspond to smaller reductions in prevalence and therefore longer elimination times. It is possible to estimate an effective field efficacy, which is the value of the individual efficacy most likely to yield an observed prevalence curve given constant transmission rates over the observation period and the antibiotic coverage. The effective field efficacy can be used to estimate elimination times and program effectiveness.

Here, we apply a mathematical transmission model to laboratory infection data from the Tanzanian portion of the Partnership for the Rapid Elimination of Trachoma trial (PRET (Stare et al., 2011)) to estimate the effective field antibiotic efficacy in an individual in this setting.

## Methods

### Clinical and laboratory results

Villages were monitored as part of a cluster-randomized trachoma treatment trial in Tanzania (the clinical trial registration number is NCT00792922) (Harding-Esch et al., 2010; Stare et al., 2011). In brief, 32 villages in Tanzania were randomized in a factorial design (1) to high (80%) and very high (90% or more) coverage with annual mass antibiotic treatment, and (2) for the application of a discontinuation rule or no use of such a rule. None of the villages had discontinued treatment during the first three years, and thus all 32 villages received treatment at baseline, 12, and 24 months. At a mass distribution, all individuals were offered a single dose of oral azithromycin (1 g in adults, and weight-based dosing designed to provide approximately 20 mg/kg to children over age 6 months; younger children were treated with topical tetracycline). The census list of the community was used to monitor coverage, and as each resident presented for treatment, treatment was observed and recorded in the treatment log by a community treatment assistant. Reported coverage includes a small fraction of children who were offered tetracycline ointment; however, the percentage of children receiving tetracycline never exceeded 8%.

All 32 villages were censused at baseline, 12, 24, and 36 months. One hundred randomly selected children aged 0–5 years were examined at baseline, and at 6, 12, 18, 24, 30 and 36 months after baseline. A dacron swab was passed 3 times over their inverted right upper conjunctiva, and processed for the presence of chlamydial DNA as previously described (Stare et al., 2011). The estimated prevalence of infection at 6, 12, 18, 24, 30, and 36 months was used to fit parameters in the stochastic transmission model. Individual level infection data were not available for all members of the population, since only a random sample of individuals was subjected to polymerase chain reaction (PCR) testing in general.

### Ethics statement

The study received ethical approval from institutional review board (IRB) of the Johns Hopkins University School of Medicine, the University of California San Francisco, and the Tanzanian National Institute for Medical Research, and was carried out in accordance with the Declaration of Helsinki. All subjects provided informed consent. The informed consent given was oral, because (1) verbal consent is the most ethical way to obtain consent, due to the high illiteracy rates in the study area, (2) IRB approved the use of the oral consent procedure for this study and (3) this oral consent is documented on the registration form for each study participant prior to examination in the field.

### Modeling methods

We modeled village chlamydial positivity rates at baseline, and at 6, 12, 18, 24, 30 and 36 months in each of 32 villages. The observed data consisted of (1) the number $S_{j}^{\left(l\right)}$ of PCR-positive individuals in the random sample with size of $M_{j}^{\left(l\right)}$ at each observation time point *l* (*l* = 0, 1, …, 6 corresponding to baseline, 6, 12, 18, 24, 30 and 36 months, respectively) for village *j* (*j* = 1, …, 32), and (2) the number of individuals reported to have been covered by antibiotics at treatment time point *k* (*k* = 1, 2, 3 corresponding to baseline, 12 and 24 months).

Because reinfection may occur following treatment, we estimated the efficacy of treatment using a stochastic transmission model of transmission of *Chlamydia trachomatis* over time, similar to models previously published (Blake et al., 2009; Lietman et al., 2011; Ray et al., 2007, 2009). We fitted this mathematical model to the infection data using the maximum likelihood method. The model contains three components: (1) random sampling of individuals for PCR testing at the observation times, (2) change in the number of infected individuals over time due to transmission and recovery, and (3) change in the number of infected individuals due to mass antibiotic treatment with the reported coverage levels (at baseline, 12 and 24 months). Observations from different villages were considered independent.

Individuals were assumed to have been sampled at random. Let *S_j_* be the number of positive individuals detected in the sample at the end of twelve months (for village *j*). From village *j* with population size *N_j_* of which the number *Y_j_* of infectives equals *i*, the probability *P*(*S_j_* = *s*|*Y_j_* = *i*) that s positives are observed from a sample of size *M_j_* is given by $\left(\begin{array}{c} i\\ s \end{array})(\begin{array}{c} N_{j}-i\\ M_{j}-s \end{array})/(\begin{array}{c} N_{j}\\ M_{j} \end{array}\right)$ using the hypergeometric distribution. For village *j* (*j* = 1, …, 32), we assumed a population of size *N_j_*, taken from the number of pre-school children found in the census at the time of treatment (at baseline, 12 or 24 months).

To model the change in prevalence between the prevalence surveys based on above assumptions, we used a classical SIS (susceptible-infective-susceptible) model structure, assuming that the force of infection is proportional to the prevalence of infection in the population with proportionality constant *β*. Moreover, we also assumed a constant exogenous force of infection *ξ* from outside the village (i.e., representing a risk which is independent of the village prevalence). Finally, we assumed a constant per-capita recovery rate *γ*. Between periods of treatment, we assumed that the probability $p_{i}^{\left(k\right)}(t)$ that there are *i* infectives in the population at time *t* after treatment time point *k* obeys the following equations for each village *j* (suppressing the subscript for clarity):

(1) $$\begin{array}{c} \frac{dp_{0}^{\left(k\right)}}{dt}=\gamma p_{1}^{\left(k\right)},\\ \frac{dp_{i}^{\left(k\right)}}{dt}=\beta \frac{\left(i-1)(N-i+1\right)}{N}p_{i-1}^{\left(k\right)}+\gamma (i+1)p_{i+1}^{\left(k\right)}-\beta \frac{i(N-i)}{N}p_{i}^{\left(k\right)}-\gamma ip_{i}^{\left(k\right)}-p_{i}^{\left(k\right)}\xi (N-i)+p_{i-1}^{\left(k\right)}\xi (N-i+1),\text{for}\;1,\leq i\leq N-1\\ \frac{dp_{N}^{\left(k\right)}}{dt}=\beta \frac{N-1}{N}p_{N-1}^{\left(k\right)}-\gamma Np_{N}^{\left(k\right)}+\xi p_{N-1}^{\left(k\right)} \end{array}$$

where *β* is the transmission coefficient, *ξ* is the risk of infection from outside the village, *γ* is the recovery rate, *i* and *N* indicate the number of infective individuals and total population of the village at time *t*, and *k* is the time point of treatment.

To model treatment, we assumed each individual in village *j* has probability $c_{j}^{\left(k\right)}$ of receiving treatment for treatment period *k* (*k* = 1, 2, 3), where $c_{j}^{\left(k\right)}$ is the probability that each child (whose age is between 0 and 5) in village *j* receives treatment in the *k*th treatment; note that treatment is assumed to occur at a specific time for everyone. The antibiotic efficacy at treatment *k* is denoted as *e_k_*. We have assumed that the probability of treatment is independent of infection status, though some evidence indicates that individuals who do not participate in mass azithromycin-based anti-trachoma campaigns may have a somewhat lower risk of infection (Amza et al., 2013; Keenan et al., 2012b).

We modeled each treatment according to $p_{i}^{\left(k\right)}(t=0)=\sum_{i^{\prime }=0}^{N_{j}}p_{i^{\prime }}^{\left(k,\text{pre}\right)}(\begin{array}{c} i^{\prime }\\ i \end{array}){\left(1-c_{j}^{\left(k\right)}e_{k}\right)}^{i}{\left(c_{j}^{\left(k\right)}e_{k}\right)}^{i^{\prime }-i}$, where *i′* is the number of infected individuals eligible for treatment, and $p_{i^{\prime }}^{\left(k,\text{pre}\right)}$ is the probability of *i* infected individuals before treatment time point *k*. For simplicity, we assumed a standard beta-binomial prior $P(Y=y)=(\begin{array}{c} N_{j}\\ y \end{array})B(y+\mu ,N_{j}-y+\rho )/B(\mu ,\rho )$ (where the shape parameters *μ* and *ρ* for each treatment were computed from the observed distribution of infection of 32 villages at baseline, 12 and 24 months, and *B*(*z*_1_, *z*_2_) is the beta function (Abramowitz and Stegun, 1972)); as a sensitivity analysis, we chose the special case *μ* = *ρ* = 1, yielding a uniform prior. The pre-treatment prevalence distribution was then computed for each village by applying Bayes' theorem:

(2) $$p_{i}^{\left(k,\text{pre}\right)}=P(Y=i|S=s)=\frac{P(S=s|Y=i)P(Y=i)}{\sum_{i=0}^{N_{j}}P(S=s|Y=i)P(Y=i)}$$

For each village *j*, we used the most recent census data to determine the village size *N_j_*. The initial condition is determined from Eq. (2), and the system numerically integrated for twelve months according to Eq. (1). Specifically, for each village *j*, the pre-treatment distributions of 1st, 2nd and 3rd treatments are $p_{i}^{\left(1,\text{pre}\right)}=P(Y=i|S=S_{j}^{\left(0\right)}),p_{i}^{\left(2,\text{pre}\right)}=P(Y=i|S=S_{j}^{\left(2\right)})$ and $p_{i}^{\left(3,\text{pre}\right)}=P(Y=i|S=S_{j}^{\left(4\right)})$ respectively (where $S_{j}^{\left(0\right)}$ is the observed number of positive individuals (before the 1st treatment) at baseline in village *j*, $S_{j}^{\left(2\right)}$ is the observed number of positive individuals (after the 1st treatment and before the 2nd treatment) at 12 month in the same village, and $S_{j}^{\left(4\right)}$ is the observed number of positive individuals (after the 2nd treatment and before the 3rd treatment) at 24 month). Given the number *i* of infected individuals, we compute the probability of the observed data of treatment *k* in village *j* according to

$$P(S_{j}=s)=\sum_{i=0}^{N_{j}}p_{i}^{\left(k\right)}(\tau )\frac{\left(\begin{array}{c} i\\ s \end{array})(\begin{array}{c} N_{j}-i\\ M_{j}-s \end{array}\right)}{\left(\begin{array}{c} N_{j}\\ M_{j} \end{array}\right)}$$

(where *M_j_* here denotes the sample size at the end of the period *k*, and *τ* is the interval between two observations). Specifically, the probability of the observed PCR-positive individuals after eachtreatment *k* in each village *j* can be represented as

$$P(S_{j}=S_{j}^{\left(2\right)})=\sum_{i=0}^{N_{j}}p_{i}^{\left(k=1\right)}(t=12)\frac{\left(\begin{array}{c} i\\ S_{j}^{\left(2\right)} \end{array})(\begin{array}{c} N_{j}-i\\ M_{j}^{\left(2\right)}-S_{j}^{\left(2\right)} \end{array}\right)}{\left(\begin{array}{c} N_{j}\\ M_{j}^{\left(2\right)} \end{array}\right)},$$

$$P(S_{j}=S_{j}^{\left(4\right)})=\sum_{i=0}^{N_{j}}p_{i}^{\left(k=2\right)}(t=12)\frac{\left(\begin{array}{c} i\\ S_{j}^{\left(4\right)} \end{array})(\begin{array}{c} N_{j}-i\\ M_{j}^{\left(4\right)}-S_{j}^{\left(4\right)} \end{array}\right)}{\left(\begin{array}{c} N_{j}\\ M_{j}^{\left(4\right)} \end{array}\right)},$$

and

$$P(S_{j}=S_{j}^{\left(6\right)})=\sum_{i=0}^{N_{j}}p_{i}^{\left(k=3\right)}(t=12)\frac{\left(\begin{array}{c} i\\ S_{j}^{\left(6\right)} \end{array})(\begin{array}{c} N_{j}-i\\ M_{j}^{\left(6\right)}-S_{j}^{\left(6\right)} \end{array}\right)}{\left(\begin{array}{c} N_{j}\\ M_{j}^{\left(6\right)} \end{array}\right)},$$

respectively (where $S_{j}^{\left(2\right)}$ and $M_{j}^{\left(2\right)}$ are observed numbers of positive individuals and sample size in village *j* at 12 month, $S_{j}^{\left(4\right)}$ and $M_{j}^{\left(4\right)}$ are observed number of positive individuals and sample size in the village at 24 month, $S_{j}^{\left(6\right)}$ and $M_{j}^{\left(6\right)}$ are observed number of positive individuals and sample size at 36 month, $p_{i}^{\left(k=1\right)}(t=12)$ is the probability that village *j* has *i* infections at 12 month (12 months after the 1st treatment at baseline), $p_{i}^{\left(k=2\right)}(t=12)$ is the probability of *i* infections at 24 month (12 months after the 2nd treatment at 12 month), and $p_{i}^{\left(k=3\right)}(t=12)$ is the probability of *i* infections at 36 month (12 months after the 3rd treatment at 24 month)). Finally, we assume independent villages, so that the total loglikelihood at time *τ* months after each treatment *k* may be computed by summing over all 32 villages $\sum_{j=1}^{32}\log(\sum_{i=0}^{N_{j}}p_{i}^{\left(k\right)}(\tau )(\begin{array}{c} i\\ s \end{array})(\begin{array}{c} N_{j}-i\\ M_{j}-s \end{array})/(\begin{array}{c} N_{j}\\ M_{j} \end{array}))$, specifically, the equation for the likelihood of three treatment periods is

$$\sum_{k=1}^{3}\sum_{j=1}^{32}\log(\sum_{i=0}^{N_{j}}p_{i}^{\left(k\right)}(\tau )\frac{\left(\begin{array}{c} i\\ S_{j}^{\left(2k\right)} \end{array})(\begin{array}{c} N_{j}-i\\ M_{j}^{\left(2k\right)}S_{j}^{\left(2k\right)} \end{array}\right)}{\left(\begin{array}{c} N_{j}\\ M_{j}^{\left(2k\right)} \end{array}\right)}).$$

### Statistical methods

We estimated the efficacy *e* for the study period, assuming a mean recovery time of 6 months (*γ* = 1/6 month^−1^); previous models have estimated the recovery time to be from 3 to 12 months (Lietman et al., 2011; Ray et al., 2007, 2009) (*β* was jointly estimated along with the efficacy; *ξ* was assumed to be zero for the base case, so no imported infection occurs). Likelihood optimization was conducted using the Nelder–Mead downhill simplex method (Nelder and Mead, 1965) as implemented in the *optim* function of the R statistical package (each optimization had at least 8 different initial values). We then estimated the efficacies *e*_1_, *e*_2_ and *e*_3_ for the first period (0–12 months), the second period (12–24 months) and the third period (24–36 months), respectively. For the base case scenario, we estimated the standard errors and confidence intervals of the estimated overall efficacy as well as the period specific estimates by using bootstrap resampling of villages. A 64-core parallel computing platform was used for bootstrap resampling (to reduce the computational costs, each of the 64 CPUs ran 6 iterations; 384 iterations were conducted). To test the hypothesis that there is no change in the efficacy over time, we first computed the efficacy for each village and time, and then used Page's *L* test of trend (Siegel and Castellan, 1988) to assess both increasing and decreasing trends.

We conducted the following sensitivity analyses. First, we varied the recovery time (1/*γ*) from 3 to 18 months, assuming no inflow of infection (*ξ* = 0). We next allowed the rate of infection from outside the community, *ξ*, to be another unknown parameter to be estimated, and assumed the same values for the recovery rate to test whether the inflow of infection has influence on the estimated efficacy. To determine whether the method for initializing the ordinary differential equations could have affected our conclusions, we repeated the analysis assuming a uniform instead of the beta-binomial prior. For each of these, we used the leave-one-out jackknife method to evaluate the standard errors (yielding slightly more conservative, i.e. larger standard errors (Efron and Tibshirani, 1993) at considerably smaller computational cost.) For a final sensitivity analysis, we modified the force of infection by including an additional nonlinear term representing departure from a linear relationship between the prevalence and risk (Lietman et al., 2011). This yields the following equation for the change in the number of infected individuals between treatments:

(3) $$\begin{array}{c} \frac{dp_{0}^{\left(k\right)}}{dt}=\gamma p_{1}^{\left(k\right)}\\ \frac{dp_{i}^{\left(k\right)}}{dt}=\beta (\frac{\left(i-1\right)}{N}+\nu_{2}{\left(\frac{i-1}{N}\right)}^{\phi +2})(N-i+1)p_{i-1}^{\left(k\right)}+\gamma (i+1)p_{i+1}^{\left(k\right)}-\beta (\frac{i}{N}+\nu_{2}{\left(\frac{i}{N}\right)}^{\phi +2})(N-i)p_{i}^{\left(k\right)}-\gamma ip_{i}^{\left(k\right)}-p_{i}^{\left(k\right)}\xi (N-i)+p_{i-1}^{\left(k\right)}\xi (N-i+1),\text{for}\;1\leq i\leq N-1,\text{and}\\ \frac{dp_{N}^{\left(k\right)}}{dt}=\beta (\frac{\left(N-1\right)}{N}+\nu_{2}{\left(\frac{N-1}{N}\right)}^{\phi +2})p_{N-1}^{\left(k\right)}-\gamma Np_{N}^{\left(k\right)}+p_{N-1}^{\left(k\right)}\xi \end{array}$$

We used Eq. (3) (with the estimates of *ν*_2_ and *ϕ* in our previous work (Lietman et al., 2011)) to estimate the efficacy assuming the presence of this non-linear term.

Finally, to predict the critical coverage level (compliance) needed for eradication within 10 years, we used the transmission parameters (*β*,*γ*) and the efficacy of the antibiotic estimated above, and varied the coverage level from 60% to 100% for all villages. Then, we simulated (100 replications for each village) the average prevalence for all villages within ten years of treatment. All computations were performed using the R statistical program (version 2.13 R Foundation for Statistical Computing, Vienna, Austria) on the RTI MIDAS cluster (http://www.epimodels.org).

## Results

**Fig. 1:**
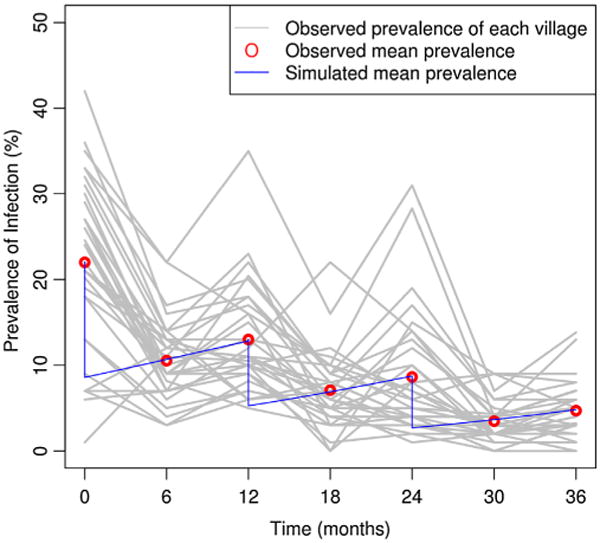
The estimated and the observed prevalence of ocular chlamydial infection in children aged 0–5 years over time. Each gray curve represents the observed prevalence in a single community over time, with 3-year treatments. The red points represent the observed mean prevalence of the 32 communities at baseline, 6, 12, 18, 24, 30 and 36 months. The blue curve shows the simulated mean prevalence based on estimates of common beta (0.228) and different effective field efficacies (64.6% at 1st treatment, 65.9% at 2nd treatment, and 76.7% at 3rd treatment) in Table 2. Oral azithromycin was distributed to communities at 0, 12 and 24 months. Each community was assessed by randomly selecting 100 children. (For interpretation of the references to color in figure legend, the reader is referred to the web version of the article.)

The numbers of children aged 0–5 at baseline, 6, 12,18, 24, 30, and 36 months tested for the presence of ocular chlamydia were 3199, 3198, 3191, 3200, 3199, 3194 and 3153, respectively. The mean of number of sampled children per village was 99.97 (SD 0.18) at baseline, 99.94 (SD 0.35) at 6-month, 99.72 (SD 0.99) at 12 months, 100 (SD 0.00) at 18 months, 99.97 (SD 0.18) at 24 months, 99.81 (SD 1.06) at 30 months, and 98.53 (SD 3.33) at 36 months. The estimated prevalence of ocular chlamydial infection by PCR at baseline was 22.0% (cover all villages), with standard deviation (SD) 10.1%. At 6 months the prevalence was 10.5% (SD 4.7%), at 12 months 13.0% (SD 6.4%), at 18 months 7.1% (SD 4.4%), at 24 months 8.6% (SD 7.3%), at 30 months 3.5% (SD 2.5%), and at 36 months 4.7% (SD 3.3%) (Fig. 1). The mean antibiotic coverage for all communities was estimated to be 93.7% (SD 5.1%) at baseline, 90.1% (SD 5.4%) at 12 months, and 89.6% (SD 4.6%) at 24 months.

Assuming that the mean duration of infection is 6 months (i.e., the recovery rate *γ* is 1/6 month^−1^) (Table 1), we found that the estimated effective field efficacy was 67.6% (95% CI: 56.5–75.1%) assuming an equal efficacy for three years. Year-specific effective field efficacy estimates are 64.6% (95% CI: 56.5–75.1%) for the first year (*ê*_1_), 65.9% (95% CI: 51.8–80.0%) for the second year (*ê*_2_), and 76.7% (95% CI: 63.5–89.8%) for the third year (*ê*_3_) (Table 2). Using Page's *L* test, we found no evidence of any change in the effective field efficacy.

Sensitivity analyses for these findings are presented in Tables 1 and 2. Assuming a recovery time of 12 months yielded a slightly higher estimated effective field efficacy for the study period: 70.7% (95% CI: 62.4–79.0%, jackknife method); we found an effective field efficacy of 72.0% (95% CI: 64.0–80.0%, jackknife method) when the mean duration of infection was 18 months and an effective field efficacy of 62.5% (95% CI: 50.1–74.9%, jackknife method) when the mean duration of infection was 3 months (Table 1). Adding an external infection term into the model yielded a slight increase (ranging from 0.02% to 2%) in the estimated effective field efficacy for all values of the mean duration of infection we examined (Table 1). We also chose a uniform prior to initialize the ordinary differential equations, and found that this yielded slightly higher estimates of the effective field efficacy as well: 71.9% (95% CI: 62.4–81.4%), 74.3% (95% CI: 65.7–83%), 75.3% (95% CI: 66.6–84.0%) and .68.1% (95% CI: 57.1%, 79.2%) for mean durations of infection of 6, 12, 18 and 3 months, respectively (confidence intervals derived by the jackknife method) (Table 1). One village (in Fig. 1) appears to have different dynamics than other 31 villages. To evaluate how sensitive the estimates in Table 1 are to that village, we used the observed data from the remaining 31 to estimate the effective field efficacy and transmission coefficient under the base case scenario, and found that the estimated effective field efficacy was 65.8% (95% CI: 55.5–74.7%, bootstrap method), and the transmission coefficient was 0.221 (95% CI: 0.197–0.256).

As a final sensitivity analysis, we added the non-linear incidence term, and found the estimated common effective field efficacies (durations of infection: 6, 12, 18 and 3 months) for three years were 66.9% (95% CI: 57.8–75.9%) with log-likelihood value of −530.682, 70.5% (95% CI: 62.3–78.6%) with log-likelihood value of −534.4659, 70.8% (95% CI: 62.6–79.4%) with log-likelihood value of −537.3769, and 60.9% (95% CI: 49.4–72.4%) with log-likelihood value of −532.483. These estimates are slightly lower than efficacies estimated with the linear term. Confidence intervals were derived using the jackknife method.

**Fig. 2:**
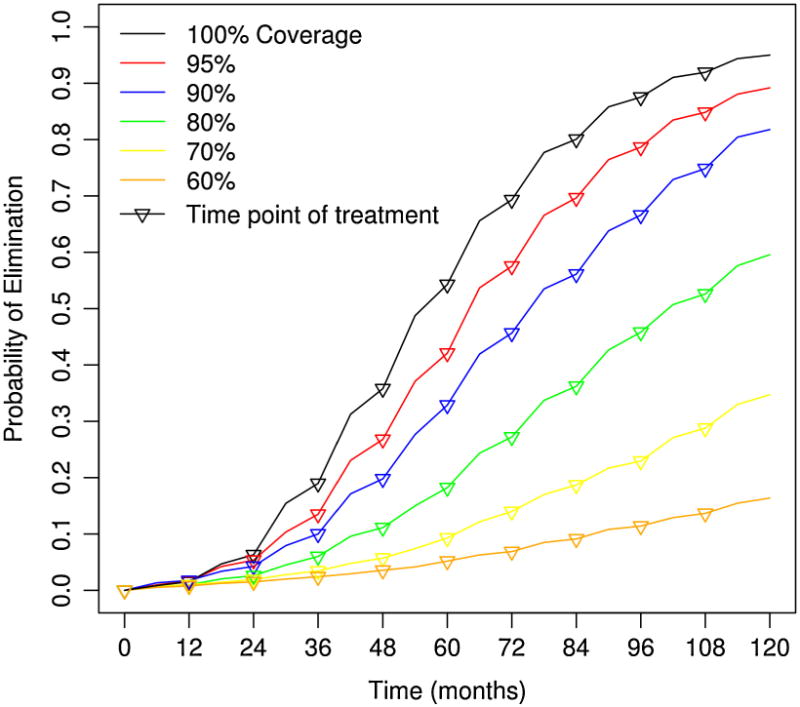
The probability of elimination by repeated mass treatment within 10 years shown for 100%, 95%, 90%, 80%, 70% and 60% coverage levels, assuming no external reintroduction of infection. Each line represents the probability of elimination happening over time for a specific antibiotic coverage using the estimated efficacy for the base case scenario (67.6% effective field efficacy, and a mean duration of infection of six months).

Based on the above estimates for the efficacy in the base case scenario, we simulated trachoma transmission with constant efficacy over times in each village to derive the probability of elimination (defined as the absence of infection and transmission). With an antibiotic efficacy of 67.6% (95% CI: 56.5–75.1%) and a mean duration of infection of 6 months, we found that the elimination probability after 10 years treatment was 95.0% (SD 4.1%) assuming complete coverage, and 89.2% (SD 5.6%) with coverage of 95%. When coverage levels of 90%, 80%, 70% and 60% were assumed, we found the probability of elimination was 81.8% (SD 8.8%), 59.6% (SD 14.8%), 34.7% (SD17.5%) and 16.4% (SD 14.3%), respectively (Fig. 2).

More generally, we may theoretically assess elimination by mass treatment by requiring sufficient coverage and efficacy that effective reproductive number *R*_eff_ = exp((*β*–*γ*)*τ*)(1 – *ec*) < 1 (where *τ* is the interval between two treatments, *c* is coverage, *β* is the transmission coefficient, 1/*γ* is the mean duration of infection, and *e* is the efficacy) (Melese et al., 2004). The estimated efficacy of 67.6% (R_eff_ = 0.68) is higher than the critical efficacy level 52.6% needed for annual treatment to eliminate infection with perfect coverage (*τ* = 12 months, 1/*γ* = 6 months, *β* = 0.229, and *c* = 100%), implying that annual mass treatment could eventually eliminate the infection.

As sensitivity analysis of different infection durations, we also simulated trachoma transmission under three 10-year treatments with 12 months infection duration and the corresponding effective field efficacy of 70.7% (95% CI: 62.4–79.0%), 18 months infection duration and corresponding efficacy of 72.0% (95% CI: 64.0–80.0%), and 3 months infection duration and the corresponding efficacy of 62.5% (95% CI: 50.1–74.9%). Assuming complete coverage, the simulated elimination probabilities after 10-year treatments were 94.1% (SD 3.1%) for infection duration of 12 months, 94.4% (SD 3.6%) for infection duration of 18 months, and 94.1% (SD 4.2%) for infection duration of 3 months. When coverage levels of 95%, 90%, 80%, 70% and 60% were assumed, we found that the probability of elimination with infection duration of 12 months was 86.8% (SD: 7.6%), 73.6% (SD 11.4%), 40.9% (SD 18.3%), 15.4% (SD 15.0%) and 4.6% (SD 10.8%), respectively (Fig. 3 in Supplement); the probability of elimination with infection duration of 18 months was 85.6% (SD: 6.5%), 70.9% (SD 12.5%), 32.3% (SD 18.4%), 10.9% (SD 15.4%) and 2.9% (SD 8.9%), respectively (Fig. 1 in the Supplement); the probability of elimination with infection duration of 3 months was 90.6.8% (SD: 5.9%), 85.3% (SD 7.9%), 71.8% (SD 12.8%), 57.3% (SD 16.8%) and 40.2% (SD 17.9%), respectively (Fig. 2 in the Supplement).

## Discussion

Applying a transmission model to data collected from a 32-village, cluster-randomized trachoma elimination trial in Tanzania, we estimated an effective field efficacy of oral azithromycin in clearing the ocular chlamydial strains that cause trachoma. Specifically, we used a transmission model to find the efficacy of oral azithromycin most likely to have resulted in the observed data pre-treatment, 6 and 12 months after each treatment, given values for the coverage. This effective field efficacy was estimated to be 67.6%. This estimate is lower than the clearance rates of 92–98% which had been reported in genital chlamydia and assumed in previous trachoma transmission models (Erdogru et al., 1995; Hillis et al., 1998; Lietman et al., 1999; Magid et al., 1996; Stamm et al., 1995; Steingrímsson et al., 1994; Thorpe et al., 1996; Wehbeh et al., 1998). More recently, the efficacy of azithromycin in clearing the sexually transmitted infection was estimated to be 77%, lower than previously thought (Handsfield, 2011; Schwebke et al., 2011).

One possible explanation for finding a lower efficacy estimate than previously found is the acquisition of macrolide resistance. If present, we would expect resistant strains to be selected for with the first mass treatment, and a progressively lower observed efficacy with each mass treatment. There were a few villages treated in previous years going back to 1999, but no Kongwa-wide mass treatment until the start of the PRET study (S. West, pers. commun.).

Our estimate may be biased for several reasons. Our base case model assumed that transmission is proportional to the number of infectious cases and number of susceptible cases (mass action); if this is not the case, then this may have masked increased transmission at the later, lower prevalence (Lietman et al., 2011). Similarly, uncertainty in either the average duration of infection or in the distribution of infection times is a potential source of model misspecification, although a sensitivity analysis suggests that efficacy estimates remain low over a wide range of assumptions. Furthermore, the Roche Amplicor test may not have detected all cases of infection, either before or after treatment (Keenan et al., 2012a; Yang et al., 2007). Even though the trial from which these data came is one of the larger trachoma studies performed, 32 communities may not be a large enough sample size to offer a precise estimate of efficacy, and the confidence interval for our estimate is broad. Loss of immunity was not considered in this model (analysis suggests a similar transmission coefficient at each follow-up period (Liu et al., 2013), contrary to expectations if loss of immunity played an important role). We also observe that if children who received antibiotics were more likely to be infected (Amza et al., 2013; Keenan et al., 2012b), then we expect that our estimates of the efficacy are biased upward (because the true probability of treatment is higher than the coverage). Our model assumed that children were treated with azithromycin only, however, some children (0–6 months) were treated with topical tetracycline, and this could be potential bias even though the percentage of children receiving tetracycline never exceeded 8%. Finally, these results are dependent on the accuracy of the census. If children were not identified and treated, the effective antibiotic coverage may have been lower than recorded, resulting in an underestimate of antibiotic efficacy. Several field control measures were instituted to ensure accurate census information, and all Community Treatment Assistant data on coverage was independently verified.

We modeled infection from outside the population of children in each community using a simple constant exogenous infection rate. Such exogenous infection may represent introduction from outside the community, or a first approximation to infection from older children or adults within the same community. Of course, such models could be further refined to reflect age structured transmission dynamics. In this setting, the other age groups (older children and adults) were being treated as well, and other studies have shown consistently higher prevalence in small children than in other age groups (e.g. Solomon et al., 2004). Previous studies have shown that treatment restricted to children can lower the prevalence in adults (House et al., 2009).

In our model, the transmission coefficient is determined by the constellation of factors which affect transmission. To the extent that transmission is reduced by the F and E components of the SAFE strategy, the estimated transmission coefficients would be smaller. Face washing (Ejere et al., 2012) and environmental sanitation (Rabiu et al., 2012; Stoller et al., 2011; West et al., 2006) for trachoma control, while less well supported by current literature than antibiotic distribution for trachoma control, could reduce the transmission coefficient and thereby enhance trachoma elimination. Estimates of the transmission coefficient in one setting could not be straightforwardly applied to other regions or times.

Models have predicted that with high coverage of an efficacious antibiotic, repeated distributions can eliminate infection from even the most severely affected communities (Lietman et al., 1999; Melese et al., 2004). Longitudinal studies have validated that local elimination is possible (Biebesheimer et al., 2009; Gaynor et al., 2003; Gill et al., 2008; Melese et al., 2008; Solomon et al., 2004). In a population that has received previous rounds of mass treatment, we found a lower efficacy of antibiotic than had been assumed. However the WHO-recommended 80% coverage and repeated rounds of treatment are projected to lead to substantial declines in trachoma prevalence and may even lead to complete elimination of infection.

## Supplementary Material

1

2

F1

F2

F3

## Figures and Tables

**Table 1:** Estimated effective field efficacy based on the data of 32 villages. We estimated the overall efficacies under the base case (6-month infection duration, no infection from outside community and beta-binomial distribution of infectious population before treatment) and sensitivity analysis scenarios of different durations of infection (6, 12, 18 and 3 months), different distributions of infectious population before treatment (beta-binomial and uniform), and different infection from outside community (included or not). The base case was done by using Bootstrap method and sensitivity analysis scenarios were done by using Jackknife method.

Scenario	Duration of infection	Distribution of infectious population before treatment	Efficacy *ê* (95% CI.)	*β̂* (95% CI.)	*ξ̂* (S.D.)	Log-likelihood value
Base Case	6-month^a^	Beta-binomial	0.676 (0.565, 0.751)	0.229 (0.202, 0.262)	–	−532.579
Varying attack duration	12-month	Beta-binomial	0.707 (0.624, 0.790)	0.146 (0.117, 0.174)	–	−535.762
	18-month	Beta-binomial	0.720 (0.640, 0.800)	0.119 (0.091, 0.143)	–	−538.468
	3-month	Beta-binomial	0.625 (0.501, 0.749)	0.401 (0.366, 0.436)	–	−535.554
Infection from outside community included (*ξ*)	6-month	Beta-binomial	0.683 (0.598, 0.768)	0.226 (0.187, 0.264)	0.0003 (9.4e–5)	−532.237
	12-month	Beta-binomial	0.707 (0.566, 0.847)	0.143 (0.102, 0.185)	0.0002 (9.8e–5)	−535.643
	18-month	Beta-binomial	0.721 (0.647, 0.796)	0.118 (0.082, 0.154)	0.0001 (8.6e–5)	−538.409
	3-month	Beta-binomial	0.645 (0.541, 0.750)	0.396 (0.353, 0.440)	0.0006 (1.0e–4)	−534.709
	6-month	Uniform	0.719 (0.624, 0.814)	0.224 (0.185, 0.264)	0.0008 (9.4e–5)	−543.023
	12-month	Uniform	0.743 (0.657, 0.830)	0.143 (0.105, 0.180)	0.0006 (8.5e–5)	−548.985
	18-month	Uniform	0.753 (0.666, 0.840)	0.117 (0.078, 0.155)	0.0006 (8.2e–5)	−552.814
3-month	Uniform	0.681 (0.571, 0.792)	0.394 (0.351, 0.438)	0.0012 (1.1e–4)	−542.268
Initialization	6-month	Uniform	0.702 (0.606, 0.798)	0.233 (0.200, 0.266)	–	−545.475
	12-month	Uniform	0.735 (0.648, 0.821)	0.151 (0.118, 0.183)	–	−550.937
	18-month	Uniform	0.747 (0.664 0.830)	0.125 (0.092, 0.157)	–	−554.559
	3-month	Uniform	0.648 (0.528, 0.768)	0.404 (0.367, 0.440)	–	−545.411

a Estimation was done by using Bootstrap method.

**Table 2:** Difference in effective field efficacy among three periods. We estimated three different effective field efficacies under scenarios of different durations of infection (6, 12, 18 and 3 months) and different distributions of infectious population before treatment (beta-binomial and uniform).

Duration of infection	Distribution of infectious population before treatment	Efficacy first period *ê*_1_ (95% C.I.)	Efficacy second period *ê*_2_ (95% C.I.)	Efficacy third period *ê*_3_ (95% C.I.)	*β̂* (95% C.I.)	Log-likelihood value
6-month	Beta-binomial	0.646 (0.565, 0.728)	0.659 (0.518, 0.800)	0.767 (0.635, 0.898)	0.228 (0.199, 0.258)	−528.235
12-month	Beta-binomial	0.687 (0.616, 0.759)	0.690 (0.563, 0.817)	0.791 (0.671, 0.912)	0.146 (0.117, 0.175)	−530.886
18-month	Beta-binomial	0.702 (0.625, 0.780)	0.703 (0.578, 0.828)	0.801 (0.677, 0.926)	0.120 (0.089, 0.150)	−533.339
3-month	Beta-binomial	0.571 (0.461, 0.683)	0.612 (0.439, 0.784)	0.726 (0.571, 0.881)	0.400 (0.366, 0.434)	−531.755
6-month	Uniform	0.667 (0.585, 0.748)	0.688 (0.551, 0.825)	0.799 (0.684, 0.913)	0.233 (0.201, 0.265)	−539.776
12-month	Uniform	0.707 (0.490, 0.923)	0.718 (0.420, 0.965)	0.821 (0.566, 0.926)	0.150 (0.080, 0.221)	−544.460
18-month	Uniform	0.727 (0.660, 0.792)	0.734 (0.612, 0.857)	0.834 (0.734, 0.935)	0.125 (0.095, 0.156)	−547.716
3-month	Uniform	0.587 (0.475, 0.698)	0.636 (0.470, 0.802)	0.755 (0.618, 0.891)	0.402 (0.367, 0.438)	−540.545
