# Supplementary material for: The efficacy of oral azithromycin in clearing ocular chlamydia: Mathematical modeling from a community-randomized trachoma trial
Source: Epidemics. Author manuscript; Available in PMC 2015 May 5. (PMC4420489; doi:10.1016/j.epidem.2013.12.001)
Supplement: 1 [file NIHMS590378-supplement-1.doc]

**Supplement:**

**Table 1.** Estimated effective field efficacy based on the data of 31 villages. We estimated the overall efficacies under the base case (6-month infection duration, no infection from outside community and beta-binomial distribution of infectious population before treatment) and sensitivity analysis scenarios of different durations of infection (6, 12, 18 and 3 months), different distributions of infectious population before treatment (beta-binomial and uniform), and different infection from outside community (included or not). The base case was done by using Bootstrap method and sensitivity analysis scenarios were done by using Jackknife method.

**Figure 1.** The probability of elimination by repeated mass treatment within 10 years shown for 100%, 95%, 90%, 80%, 70% and 60% coverage levels, assuming no external reintroduction of infection. Each line represents the probability of elimination happening over time for a specific antibiotic coverage using the estimated efficacy for the varying attack duration scenario (72.0% effective field efficacy, and a mean duration of infection of eighteen months).

**Figure 2.** The probability of elimination by repeated mass treatment within 10 years shown for 100%, 95%, 90%, 80%, 70% and 60% coverage levels, assuming no external reintroduction of infection. Each line represents the probability of elimination happening over time for a specific antibiotic coverage using the estimated efficacy for the varying attack duration scenario (62.5% effective field efficacy, and a mean duration of infection of three months).

**Figure 3.** The probability of elimination by repeated mass treatment within 10 years shown for 100%, 95%, 90%, 80%, 70% and 60% coverage levels, assuming no external reintroduction of infection. Each line represents the probability of elimination happening over time for a specific antibiotic coverage using the estimated efficacy for the varying attack duration scenario (70.7% effective field efficacy, and a mean duration of infection of twelve months).
