# Supplementary material for: The efficacy of oral azithromycin in clearing ocular chlamydia: Mathematical modeling from a community-randomized trachoma trial
Source: Epidemics. Author manuscript; Available in PMC 2015 May 5. (PMC4420489; doi:10.1016/j.epidem.2013.12.001)
Supplement: 2 [file NIHMS590378-supplement-2.doc]

**Supplement:**

**Table 1.** Estimated effective field efficacy based on the data of 31 villages. We estimated the overall efficacies under the base case (6-month infection duration, no infection from outside community and beta-binomial distribution of infectious population before treatment) and sensitivity analysis scenarios of different durations of infection (6, 12, 18 and 3 months), different distributions of infectious population before treatment (beta-binomial and uniform), and different infection from outside community (included or not). The base case was done by using Bootstrap method and sensitivity analysis scenarios were done by using Jackknife method.

| **Scenario** | **Duration of infection** | **Distribution of infectious population before treatment** | **Efficacy ê (95% C.I.)** | $\hat{\beta}$ **(95% C.I.)** | $\hat{\xi}$ **(S.D.)** |
| --- | --- | --- | --- | --- | --- |
| **Base Case** | 6-month* | Beta-binomial | 0.658 (0.555, 0.747) | 0.221 (0.197, 0.256) | - |
| **Varying attack duration** | 12-month | Beta-binomial | 0.698 (0.607, 0.788) | 0.140 (0.112, 0.168) | - |
|  | 18-month | Beta-binomial | 0.713 (0.626, 0.800) | 0.114 (0.086, 0.142) | - |
|  | 3-month | Beta-binomial | 0.593 (0.468, 0.718) | 0.390 (0.360, 0.419) | - |
| **Infection from outside community included ($\xi$)** | 6-month | Beta-binomial | 0.674 (0.577, 0.770) | 0.213 (0.179, 0.247) | 0.0007 (7.6e-5) |
|  | 12-month | Beta-binomial | 0.706 (0.617, 0.794) | 0.133 (0.099, 0.168) | 0.0005 (7.1e-5) |
|  | 18-month | Beta-binomial | 0.719 (0.635, 0.803) | 0.108 (0.074, 0.143) | 0.0005 (7.3e-5) |
|  | 3-month | Beta-binomial | 0.627 (0.511, 0.743) | 0.381 (0.346, 0.416) | 0.0011 (8.4e-4) |
|  | 6-month | Uniform | 0.710 (0.602, 0.819) | 0.212 (0.185, 0.264) | 0.0012 (8.0e-5) |
|  | 12-month | Uniform | 0.740 (0.638, 0.841) | 0.132 (0.096, 0.168) | 0.0010 (7.4e-5) |
|  | 18-month | Uniform | 0.752 (0.655, 0.850) | 0.107 (0.070, 0.144) | 0.0009 (7.8e-5) |
|  | 3-month | Uniform | 0.666 (0.544, 0.789) | 0.379 (0.344, 0.413) | 0.0016 (1.1e-4) |
| **Initialization** | 6-month | Uniform | 0.687 (0.584, 0.791) | 0.225 (0.193, 0.256) | - |
|  | 12-month | Uniform | 0.727 (0.632, 0.823) | 0.145 (0.112, 0.178) | - |
|  | 18-month | Uniform | 0.742 (0.651, 0.834) | 0.120 (0.086, 0.153) | - |
|  | 3-month | Uniform | 0.620 (0.496, 0.744) | 0.393 (0.361, 0.424) | - |

*Estimation was done by using Bootstrap method.
