# Supplementary figures and images for: The efficacy of oral azithromycin in clearing ocular chlamydia: Mathematical modeling from a community-randomized trachoma trial
Source: Epidemics. Author manuscript; Available in PMC 2015 May 5. (PMC4420489; doi:10.1016/j.epidem.2013.12.001)

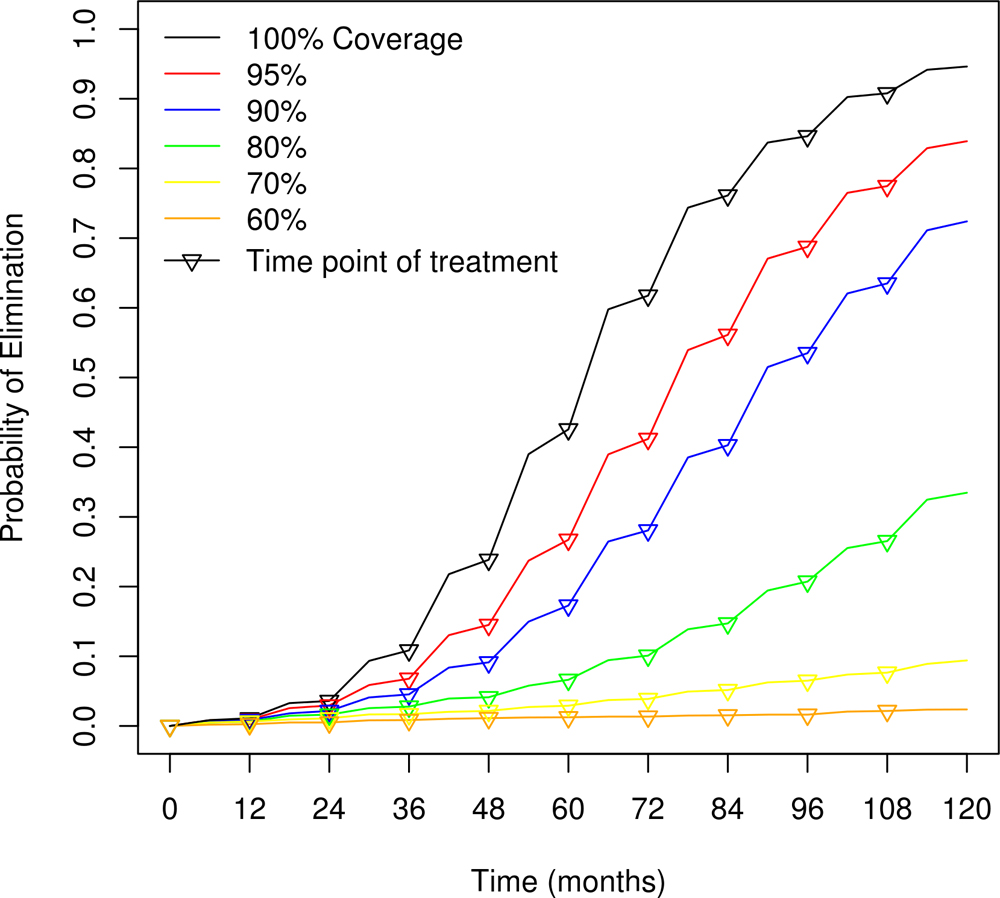

Supplement: F1 [file NIHMS590378-supplement-F1.jpg]

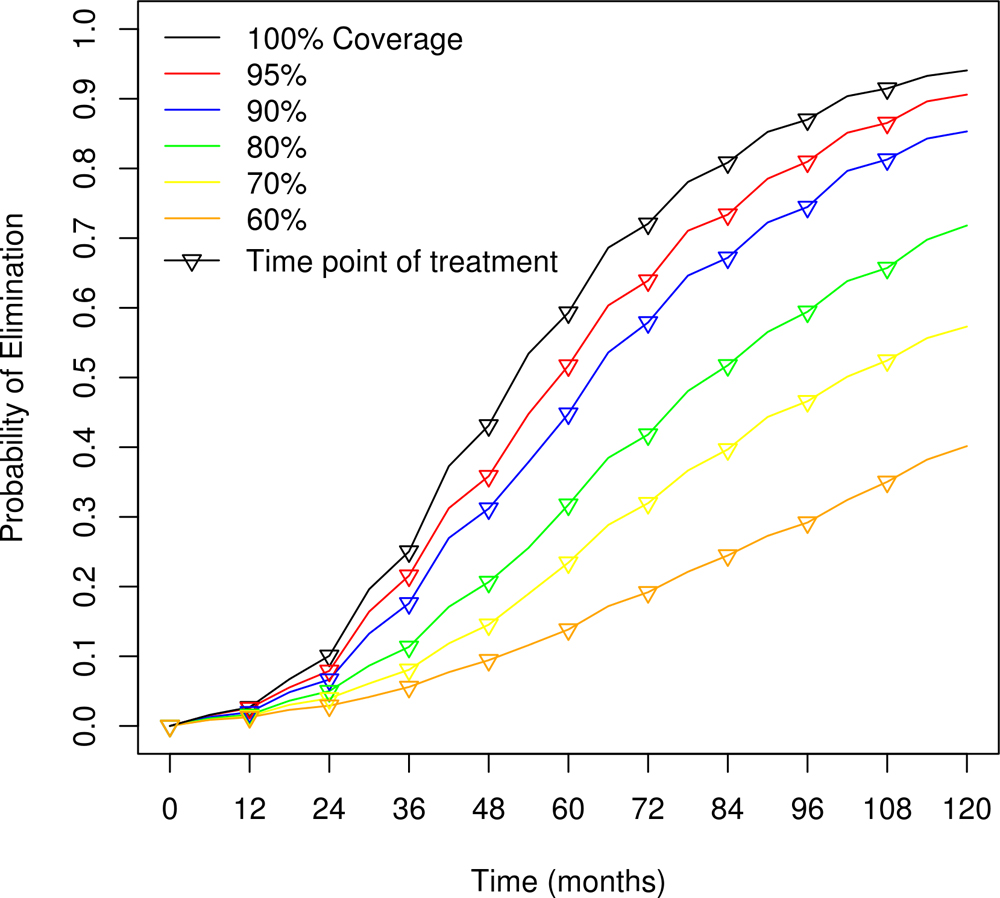

Supplement: F2 [file NIHMS590378-supplement-F2.jpg]

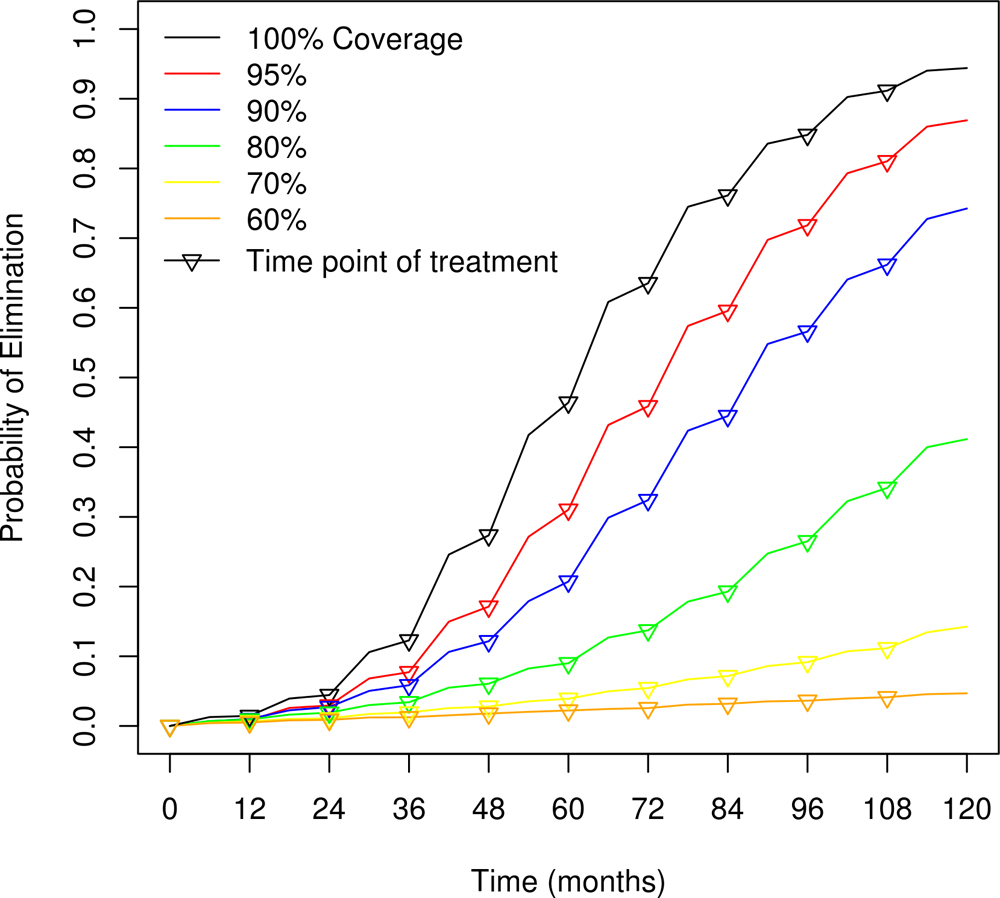

Supplement: F3 [file NIHMS590378-supplement-F3.jpg]
